# Supplementary material for: Chromothripsis during telomere crisis is independent of NHEJ, and consistent with a replicative origin
Source: Genome Res. 2019 May;29(5):737–49. doi: 10.1101/gr.240705.118 (PMC6499312; doi:10.1101/gr.240705.118)
Supplement: Supplemental Material [file supp_gr.240705.118_Supplemental_file_1.zip › contigs/annotated_contigs/DB49/contig.2.DB49_length_560_mean_cov_9.375.docx]

**DB49_length_560_mean_cov_9.375**

```
TGACTGTTTAACACCCTTACTATGACCTCACTACCTACTTTATTCTCAGATGTTCATTGTTATCTTCACCTCCTTATCTTTATCCTCAA
 >chr1:105956397-105956654 - E=3e-138
TTTCCTGTGTATTTACTTTATTTACTTAGGAAACAACCTCCAAATCTAGCTAAACTTACTTAAAAAAAATGAAACTCTCTCGAATAAAT

TCTTTTGAAATGAAAGCAAACCATAGTAATCGGTTTTATTTTCATTTATGACAACAAAACTCAAGAAGCCCTAAAGC|T|TTCCAAACT
                                                                              >chr1:105
TTTATGCTCTGCTTCCCTTTTAAAACTGAATGCCTTTAACAGCACCTAAGTCACTCCTTGAACACTTTGTCACTTAGAAATTTCTTCTG
953482-105953787 - E=7e-169
CCTGATACCCTAAATTATCTCTGTCAAGTTTAAACAAATCTCTAGGGCAGGGGCAAAATGCCACCAGACTCTTTGCTCAAACATAACAA

GACTCACCTTTGTTCTAGTTCCCAATGAGTTCCTCATTTCCATCTGAGACCACCTCAGCATGGACTTTATTGTCCATATCACTATCAGC

AATTTGGGCAAGCCCATTCAACAAGTCT
```
